# Untargeted Metabolomics Unravel the Effect of SlPBB2 on Tomato Fruit Quality and Associated Plant Metabolism

**DOI:** 10.3390/metabo16010068

**Published:** 2026-01-12

**Authors:** Cuicui Wang, Lihua Jin, Daqi Fu, Weina Tian

**Affiliations:** 1 College of Biology Engineering, Beijing Polytechnic University, Beijing 100176, China; 103094@bpu.edu.cn (C.W.); jinlihua@bpu.edu.cn (L.J.); 2 Fruit Biology Laboratory, College of Food Science and Nutritional Engineering, China Agricultural University, Beijing 100083, China; daqifu@cau.edu.cn

**Keywords:** proteasomes subunit, metabolomics, fruit quality

## Abstract

**Background**: Proteasomes are protein complexes that mediate proteolysis to degrade unneeded or damaged proteins, and they play an indispensable role in plant growth and development. However, their regulatory effects on tomato fruit quality and the underlying metabolic mechanisms remain largely elusive. This study aimed to elucidate the metabolic regulatory mechanisms of proteasomes in tomato fruits through untargeted metabolome analysis. **Methods**: An untargeted metabolomics approach was employed to profile the metabolic changes in tomato fruits. Metabolites were detected and identified under both positive and negative ion modes. Metabolic profiles were compared between wild-type (WT) tomato fruits and *SlPBB2* RNA interference (*SlPBB2*-RNAi) lines. Specifically, the *SlPBB2*-RNAi line refers to a transgenic tomato line constructed via Agrobacterium-mediated transformation, where the expression of the proteasome component gene *SlPBB2* was stably downregulated by RNA interference technology to clarify its regulatory role in fruit metabolism. KEGG enrichment analysis was performed to annotate the functions of differential metabolites. **Results**: A total of 568 and 333 metabolites were identified in positive and negative ion modes, respectively. Comparative analysis revealed 43 differentially abundant metabolites between WT and *SlPBB2*-RNAi fruits, including D-glucose, pyruvic acid, leucine, and naringenin. KEGG enrichment analysis further identified key metabolites involved in the carbon fixation pathway of photosynthetic organisms, with L-malic acid being a prominent representative. Reduced accumulation of D-glucose and pyruvic acid in *SlPBB2*-RNAi fruits suggested the inhibition of the citrate cycle, a core pathway in cellular energy metabolism. This metabolic perturbation was associated with decreased chlorophyll content in *SlPBB2*-RNAi plants, implying impaired photosynthetic carbon fixation and energy metabolism. **Conclusions**: This study uncovers the metabolic regulatory role of SlPBB2-mediated proteasome function in tomato fruits, providing novel insights into the link between proteasomal activity and fruit metabolic homeostasis from a metabolomic perspective. These findings offer new theoretical foundations for developing strategies to improve tomato nutritional quality.

## 1. Introduction

Tomato (*Solanum lycopersicum* L.) is one of the most widely consumed fresh vegetables globally, valued for its rich content of essential nutrients, antioxidants, and phytochemicals that contribute to human health. Specifically, tomato fruits are rich in bioactive components including enzymes, vitamins, sugars, monounsaturated fatty acids (linoleic and linolenic acids), amino acids, phenolics, carotenoids, and flavonoids, which collectively define their nutritional and commercial importance [1]. As a staple in daily diets, improving tomato fruit quality—encompassing nutritional value, aroma, taste, and health-promoting properties—has long been a key focus in horticultural and agricultural research, given its direct implications for consumer health and market competitiveness.

Fruit quality and nutritional value are largely determined by metabolite composition at harvest. Among these metabolites, antioxidants and health-beneficial compounds such as vitamin C, vitamin E, lycopene, β-carotene, lutein, and flavonoids (e.g., quercetin) are particularly critical, as they are closely associated with reduced risks of chronic diseases in humans [2]. Previous studies have shown that variations in early fruit development and metabolite composition can significantly influence the taste and overall quality of ripe tomatoes, highlighting the importance of understanding the regulatory mechanisms underlying fruit metabolic homeostasis [3].

The ubiquitin–proteasome system (UPS) is widely recognized as a central regulatory pathway governing nearly all aspects of plant growth and development [4,5], including seed size determination, cell signal transduction, programmed cell death, responses to biotic and abiotic stresses, circadian rhythms, and developmental transitions [6]. Within the UPS, proteasomes—multisubunit protein complexes responsible for degrading unneeded or damaged proteins via proteolysis—play a pivotal role in maintaining cellular protein homeostasis. Recent advances in tomato research have revealed that silencing the proteasome subunit PBB2 can delay fruit ripening [7], a process tightly linked to changes in fruit quality. However, despite growing knowledge of PBB2’s role in ripening, its specific impact on tomato nutritional quality and associated metabolic changes remains largely unexplored, representing a significant knowledge gap in the field [8].

Notably, although the UPS has been extensively studied in plant growth and stress responses, there is limited consensus on how individual proteasome subunits regulate specific fruit quality traits. Controversies persist regarding the tissue-specific functions of proteasome subunits: some studies suggest that proteasome-mediated regulation is highly conserved across plant organs [9], while others propose organ-specific regulatory networks that may differ between vegetative tissues and reproductive organs (e.g., fruits) [10]. This discrepancy underscores the need for targeted investigations into proteasome subunits in fruit-specific contexts.

Metabolomics has emerged as a powerful tool in systems biology, enabling the comprehensive profiling and quantification of small-molecule metabolites in biological samples. This approach facilitates the identification of metabolic changes induced by genetic modifications or environmental treatments, thereby providing insights into underlying physiological and biochemical processes in plants. Given the lack of research on PBB2 in tomato nutritional quality, high-resolution untargeted metabolomics offers an ideal strategy to elucidate metabolic perturbations associated with *PBB2* silencing.

In this study, we aimed to investigate the effect of the proteasome subunit PBB2 on tomato fruit quality and related metabolic pathways using high-resolution untargeted metabolomics. Our findings demonstrate that *PBB2* silencing leads to significant alterations in key metabolic pathways, including those involved in carbon fixation and energy metabolism, thereby influencing tomato nutritional quality. This study addresses the existing knowledge gap regarding the link between proteasome function and fruit nutritional quality and provides novel theoretical support for developing strategies to improve tomato nutritional value.

## 2. Materials and Methods

### 2.1. Plant Materials and Growth Conditions

Tomato plants (*Solanum lycopersicum* cv. Ailsa Craig) were cultivated under controlled greenhouse conditions. Plants were divided into two groups: the wild-type (WT) group and the *PBB2*-RNAi lines, with 6 biological replicates in each group (total ≥ 36 fruit). *PBB2*-RNAi transgenic plants were generated as previously described [7].

Fruit was harvested at the red-ripe stage for metabolomic analysis. Flowers were tagged at anthesis, and fruits subsequently were harvested at 43 days post-anthesis (DPA). All samples were immediately frozen in liquid nitrogen and stored at −80 °C until metabolite extraction.

### 2.2. Measurement of Total Chlorophyll

Total chlorophyll content was measured in fruits at the immature green stage (approximately 30 DPA). Chlorophyll was extracted from 2 mg of pericarp tissue using 80% (*v*/*v*) acetone at 4 °C overnight in darkness. The concentrations of chlorophyll a and b were detected by spectrophotometer and calculated according to the previous study [11]. The sum of chlorophyll a and b represented the total chlorophyll. Three biological replicates were analyzed for each genotype.

### 2.3. Metabolite Extraction

Frozen tomato fruit tissue (80 mg) was ground to a fine powder with a mortar and pestle. Metabolites were extracted with 1000 μL of pre-chilled methanol/acetonitrile/water solution (2:2:1, *v*/*v*/*v*). The homogenate was vortexed vigorously, sonicated in an ice-water bath for 30 min, and then incubated at −20 °C for 10 min. After centrifugation at 14,000× *g* for 20 min at 4 °C, the supernatant was collected and dried in a vacuum centrifuge. For LC-MS analysis, the dried residue was reconstituted in 100 μL of acetonitrile/water (1:1, *v*/*v*) and vortexed, followed by centrifugation at 14,000× *g* for 15 min at 4 °C. The resulting supernatant was transferred to an injection vial for subsequent analysis.

### 2.4. LC-MS/MS Analysis

Chromatographic separation was performed on an Agilent 1290 Infinity II UHPLC system equipped with a Waters ACQUITY UPLC BEH C18 column (1.7 μm, 2.1 mm × 100 mm). The column temperature was maintained at 40 °C. The mobile phase consisted of (A) water containing 25 mM ammonium acetate and 0.5% formic acid, and (B) methanol. The following gradient elution program was used at a flow rate of 0.4 mL/min: 0–0.5 min, 5% B; 0.5–10 min, linear increase from 5% to 100% B; 10.0–12.0 min, 100% B; 12.0–12.1 min, linear decrease to 5% B; 12.1–16 min, 5% B for column re-equilibration. The injection volume was 2 μL. Samples were maintained at 4 °C in the autosampler during analysis. To minimize instrumental bias, samples were analyzed in randomized order. Quality control (QC) samples, prepared by pooling aliquots from all samples, were injected at regular intervals to monitor system stability and data reproducibility.

Mass spectrometry detection was conducted on an AB Sciex Triple TOF 6600 system (SCIEX, Framingham, MA, USA) equipped with an electrospray ionization (ESI) source. The source parameters were set as follows: ion source gas 1 (GS1) = 60 psi, ion source gas 2 (GS2) = 60 psi, curtain gas (CUR) = 30 psi, source temperature = 600 °C, and ion spray voltage floating (ISVF) = ±5500 V. Data were acquired in both positive and negative ionization modes. The full-scan mass range was *m*/*z* 60–1000, and the product ion scan range was *m*/*z* 25–1000. Accumulation times were set to 0.20 s/spectrum for TOF-MS scans and 0.05 s/spectrum for product ion scans.

### 2.5. Data Processing and Analysis

Raw mass spectrometry data were converted to .mzXML format using ProteoWizard software (version 3.0.20229; http://proteowizard.sourceforge.net/) and then processed with MS-DIAL software (version 4.90) for peak detection, alignment, retention time correction, and peak area extraction. Metabolite identification was performed by matching accurate mass, retention time, and MS/MS fragmentation patterns against the MassBank and HMDB databases. All putative identifications were manually verified.

Differentially abundant metabolites (DAMs) between WT and *PBB2*-RNAi groups were identified by combining univariate and multivariate statistical analyses. Univariate analysis included fold-change calculation and Student’s *t*-test. Multivariate analyses, including principal component analysis (PCA), partial least squares-discriminant analysis (PLS-DA), and orthogonal PLS-DA (OPLS-DA), were performed to assess group separation and identify discriminative metabolites. Metabolites with a variable importance in projection (VIP) score > 1.0 from the OPLS-DA model and a *p*-value < 0.05 from the *t*-test were considered statistically significant DAMs.

Biological interpretation was conducted via Kyoto Encyclopedia of Genes and Genomes (KEGG) pathway annotation and enrichment analysis of the DAMs. Hierarchical clustering and correlation analyses were performed to visualize metabolite accumulation patterns and inter-metabolite relationships across samples. All statistical analyses were carried out using R software (version 4.2.1) with relevant packages.

## 3. Results

### 3.1. Silencing of SlPBB2 Reduces Chlorophyll Accumulation in the Tomato Fruit Shoulder Region

**Figure 1:**
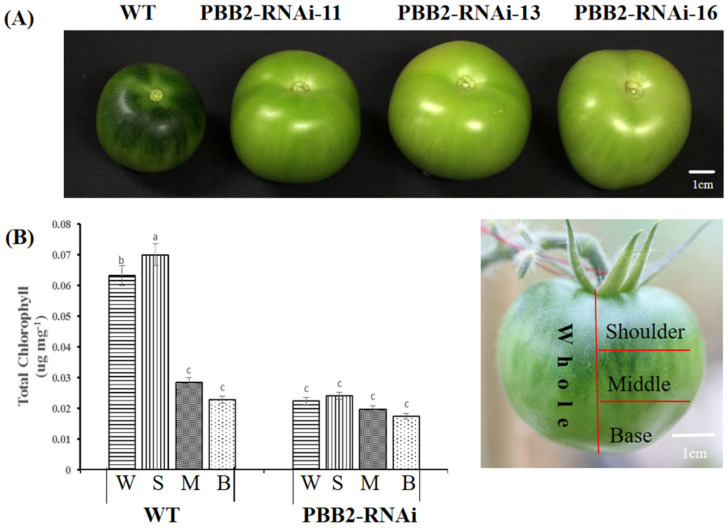
Fruit phenotype and chlorophyll content in the *PBB2* RNAi. (**A**) Variation in fruit phenotype between Ailsa Craig wild type (WT) and *PBB2*-RNAi at 30 days post-anthesis. Scale bar, 1 cm. (**B**) Chlorophyll content in the pericarp isolated from the whole (W), shoulder (S), middle (M) and base (B) of Ailsa Craig wild-type (WT) and *PBB2*-RNAi fruit at 30 days post-anthesis. Values are presented as means ± standard deviation (SD) of three independent biological replicate. Different letters indicate significant differences (*p* < 0.05) relative to the WT values by Duncan’s multiple range test.

Compared to the wild-type (WT), *PBB2*-RNAi fruits lacked the characteristic green shoulder around the calyx end during development (Figure 1A). This observation is consistent with previous findings that *SlPBB2* silencing leads to reduced chlorophyll levels in the peel and impairs chloroplast development, resulting in bigger cell sizes and fewer chloroplasts per cell. Chlorophyll accumulation is a well-established indicator of fruit maturity and quality [12]. To further elucidate the metabolic alterations and key metabolites underlying the effect of PBB2 on chlorophyll synthesis in tomato fruits, we conducted untargeted metabolomics, which provides novel insights into the associated regulatory mechanisms.

### 3.2. Quality Control (QC) of the Mass Spectrometry Data

To ensure data reliability, we comprehensively evaluated instrumental stability, experimental repeatability, and overall data quality. The total ion chromatograms (TICs) of quality control (QC) samples exhibited substantial overlap, indicating consistent chromatographic peak responses and retention times throughout the analytical sequence (Figure S1), which confirms the instrument was in a stable state during analysis.

**Figure 2:**
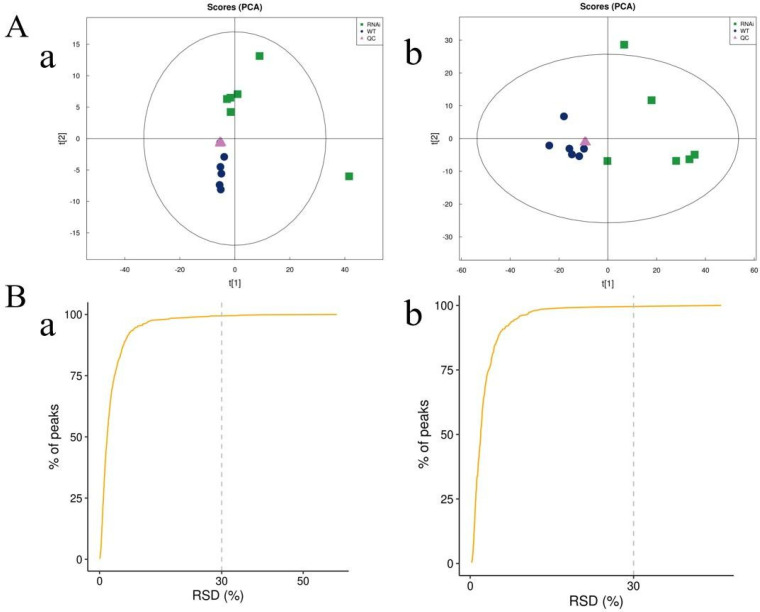
Quality control assessment of metabolomics data. (**A**) PCA score plots of QC samples in (**a**) positive and (**b**) negative ion modes. t[1] and t[2] represent principal component 1 and 2, respectively. The ellipse indicates the 95% confidence interval. Dots of the same color represent individual biological replicates, and their distribution reflects inter− and intra−group variation. (**B**) Distribution of relative standard deviation (RSD) for peaks in QC samples from (**a**) positive and (**b**) negative ion modes.

Principal component analysis (PCA) further demonstrated that QC samples clustered tightly in both positive and negative ion modes (Figure 2A), reflecting high experimental repeatability. A critical metric for evaluating instrumental stability is the relative standard deviation (RSD) of ion peak abundances in QC samples. In this study, over 80% of the peaks detected in QC samples exhibited an RSD ≤ 30% (Figure 2B), demonstrating excellent analytical system stability and validating the acquired data for subsequent statistical analyses.

### 3.3. Chemical Classification and Attribution Statistics of Metabolites

Untargeted metabolomics identified a total of 901 metabolites in tomato fruit tissues, with 568 and 333 metabolites detected in positive and negative ion modes, respectively. These metabolites encompassed a broad spectrum of chemical classes, including organic acids, amino acids, nucleotides, carbohydrates, lipids, and secondary metabolites such as flavonoids and alkaloids.

**Figure 3:**
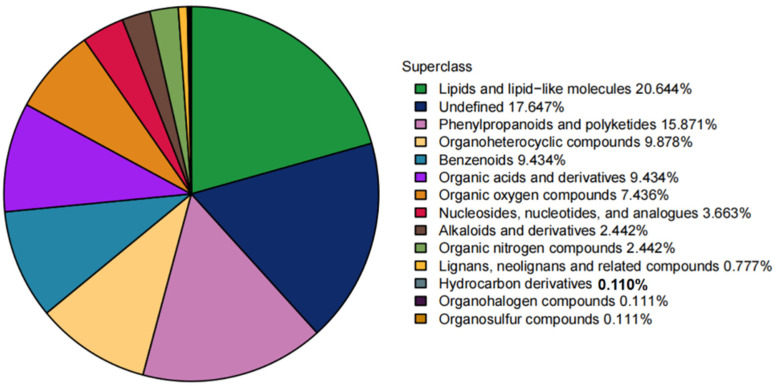
Quantitative distribution of identified metabolites across chemical classes. Different colored segments represent distinct chemical classification categories. Percentages indicate the proportion of metabolites within each category relative to the total number of identified metabolites.

Classification based on chemical taxonomy revealed that the identified metabolites were predominantly distributed among lipids and lipid-like molecules, phenylpropanoids and polyketides, and organoheterocyclic compounds (Figure 3). Metabolites lacking a defined chemical classification were categorized as “undefined”. Metabolite qualitative and quantitative results are provided in Supplementary Table S1.

### 3.4. Multivariate Analysis of Identified Metabolites and Screening of the Differential Metabolites

**Figure 4:**
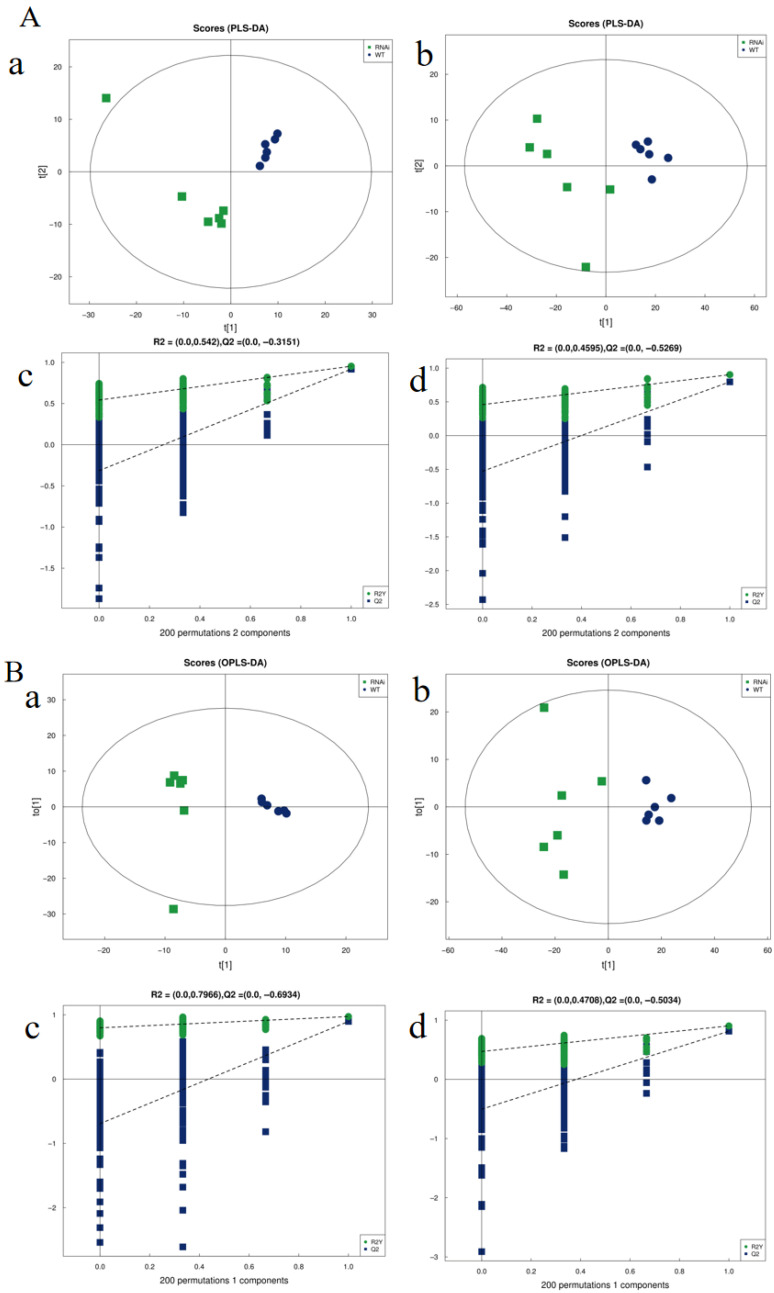
Multivariate statistical analysis. (**A**) PLS-DA score plots for (**a**) positive and (**b**) negative ion modes. Corresponding permutation test results for (**c**) positive and (**d**) negative ion modes. (**B**) OPLS-DA score plots for (**a**) positive and (**b**) negative ion modes. Corresponding permutation test results for (**c**) positive and (**d**) negative ion modes.

PCA score plots revealed clear separation between samples from the WT and *PBB2*-RNAi groups, indicating substantial differences in their metabolite profiles. Supervised multivariate analyses, including partial least squares-discriminant analysis (PLS-DA) and orthogonal projections to latent structures discriminant analysis (OPLS-DA), further confirmed this distinct separation (Figure 4A,B).

The PLS-DA models for positive and negative ion modes showed high explanatory and predictive power, with R^2^Y values of 0.953 and 0.903, and Q^2^ values of 0.918 and 0.796, respectively (Figure 4A). All Q^2^ values exceeded the acceptable threshold of 0.5. Permutation tests (n = 200) yielded R^2^ and Q^2^ intercepts that met validation criteria (R^2^ intercept < 0.3–0.4, Q^2^ intercept < 0), confirming model robustness and the absence of overfitting (Figure 4A(c,d)).

Similarly, OPLS-DA models exhibited strong performance, with R^2^Y and Q^2^ values of 0.972/0.896 (positive mode) and 0.903/0.813 (negative mode) (Figure 4B). Corresponding permutation tests also validated these models. Collectively, these results confirm that the established models effectively discriminate between the two groups with high stability and predictive accuracy.

**Figure 5:**
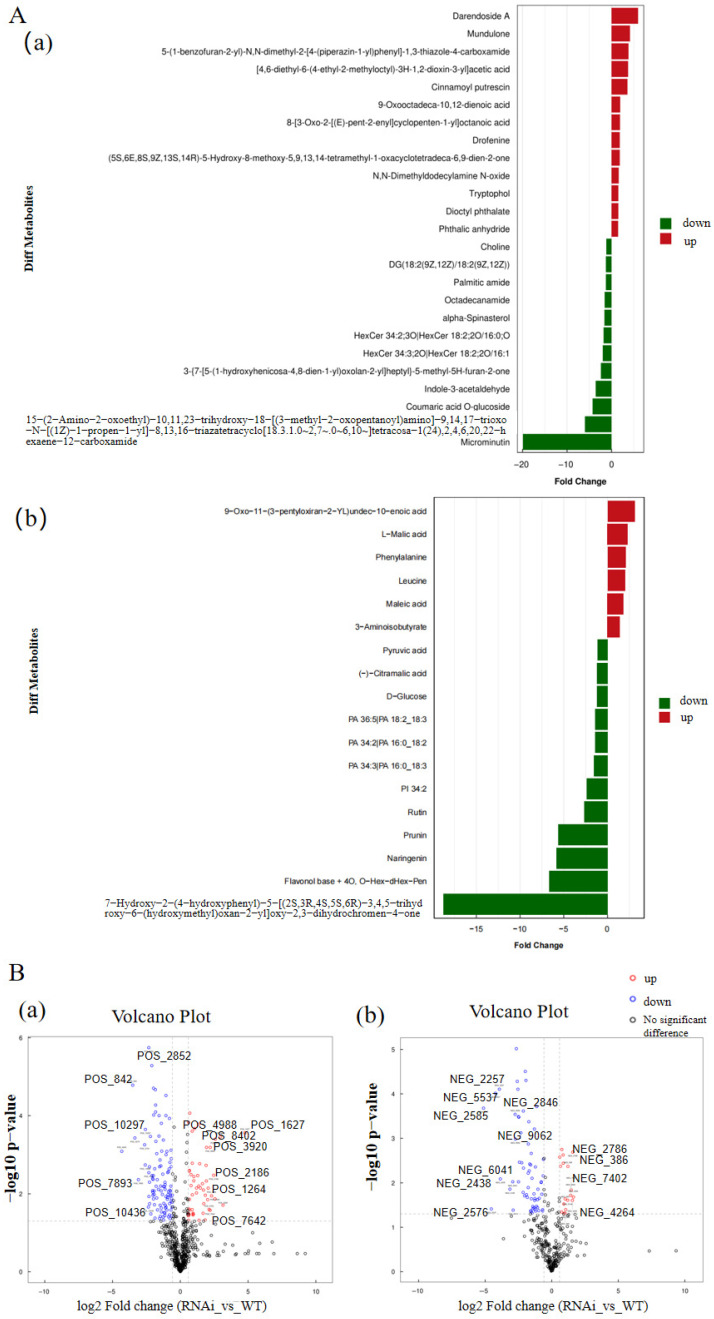
Visualization of differential metabolites. (**A**) Bar charts showing the fold-change of significantly different metabolites in (**a**) positive and (**b**) negative ion modes. (**B**) Volcano plots of all detected metabolites in (**a**) positive and (**b**) negative ion modes. Differential metabolites were defined by fold-change (FC) > 1.5 or FC < 0.67 and *p*-value < 0.05. Red dots indicate upregulated metabolites, blue dots indicate downregulated metabolites, and gray dots represent non-significant metabolites.

Differential metabolites were screened by applying thresholds of variable importance in projection (VIP) ≥ 1.0 from the OPLS-DA models and a Student’s *t*-test *p*-value < 0.05. This analysis identified 43 significant differential metabolites (Table S2). In the positive ion mode, 13 metabolites were upregulated and 12 were downregulated. In the negative ion mode, 6 were upregulated and 12 were downregulated. The differential expression patterns are visualized in volcano plots (Figure 5). These 43 key metabolites primarily belong to 14 classes, with phenylpropanoids, flavonoids, and phenolic acids being the most prominent (Figure 5).

### 3.5. Bioinformatics Analysis of Differential Metabolites

**Figure 6:**
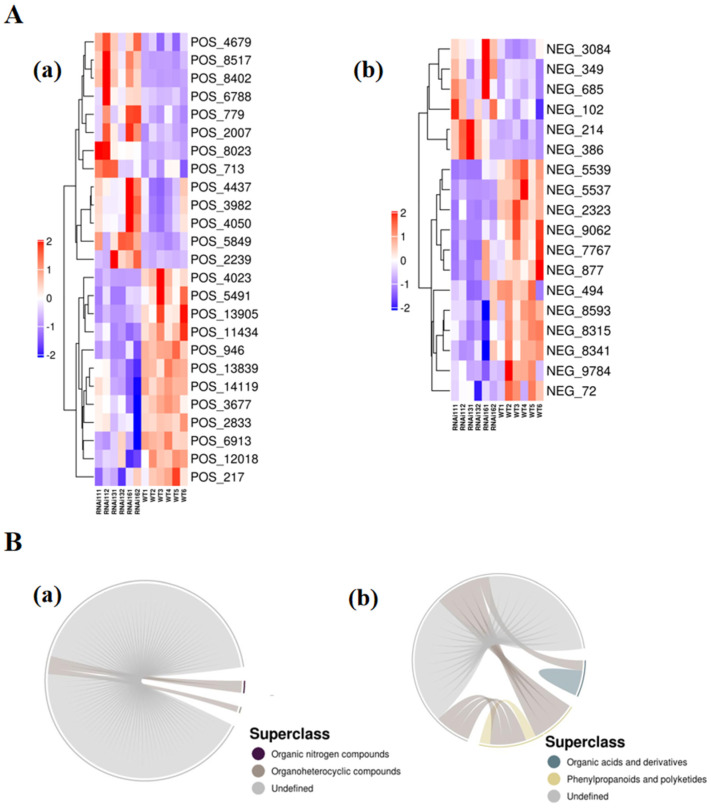
Cluster and correlation analysis of significant differential metabolites. (**A**) Hierarchical clustering heatmaps of significant differential metabolites in (**a**) positive and (**b**) negative ion modes. Each row represents a metabolite, and each column represents a sample group. Red and blue indicate upregulation and downregulation, respectively, with color intensity reflecting the degree of change. (**B**) Chord diagrams visualizing significant correlations (|r| > 0.8, *p* < 0.05) among metabolites in (**a**) positive and (**b**) negative ion modes. The inner circle represents individual metabolites, the outer arcs represent metabolite classes, and colored links denote correlations, with dark gray lines indicating inter-class correlations.

Follow-up biogenic analysis, including cluster analysis, correlation analysis and pathway analysis, was conducted for the metabolites with significant differences (metabolites should simultaneously satisfy OPLS-DA VIP > 1 and *p* value < 0.05, and have qualitative names). The hierarchical cluster analysis results of metabolites with significant differences (VIP > 1, *p* value < 0.05) are shown in Figure 6. Metabolites clustered in the same cluster have similar expression patterns and may have similar functions or participate in the same metabolic process or cellular pathway. In order to more intuitively reveal the co-regulatory relationship between various metabolites, the correlation matrix was converted into a chord diagram, as shown in Figure 6. The chord diagram shows molecular pairs of metabolites with correlation coefficient |r| > 0.8 and *p* < 0.05 [13]. The chord diagram can better show the correlation between various metabolites.

Hierarchical clustering analysis of the samples showed that the samples within the same group were clustered together, indicating high similarity in metabolite profiles within the group. The clustering analysis of differential metabolites revealed that metabolites with similar expression patterns were grouped together, suggesting potential similarities in their functions or metabolic pathways.

Correlation analysis indicated that some differential metabolites were positively correlated, while others were negatively correlated. The correlation heatmaps, chord diagrams, and network diagrams provided a visual representation of the relationships between metabolites, helping to understand the co-regulation and interaction of metabolites in the metabolic network.

### 3.6. KEGG Enrichment Analysis of Differential Metabolites

We annotated the 43 differential metabolites using the KEGG database. Most metabolites were mapped to general metabolic and biosynthetic pathways, as expected (Table S1). Subsequent enrichment analysis identified eight specialized plant metabolic pathways that were significantly altered (*p* < 0.05) between the WT and *PBB2*-RNAi groups.

**Figure 7:**
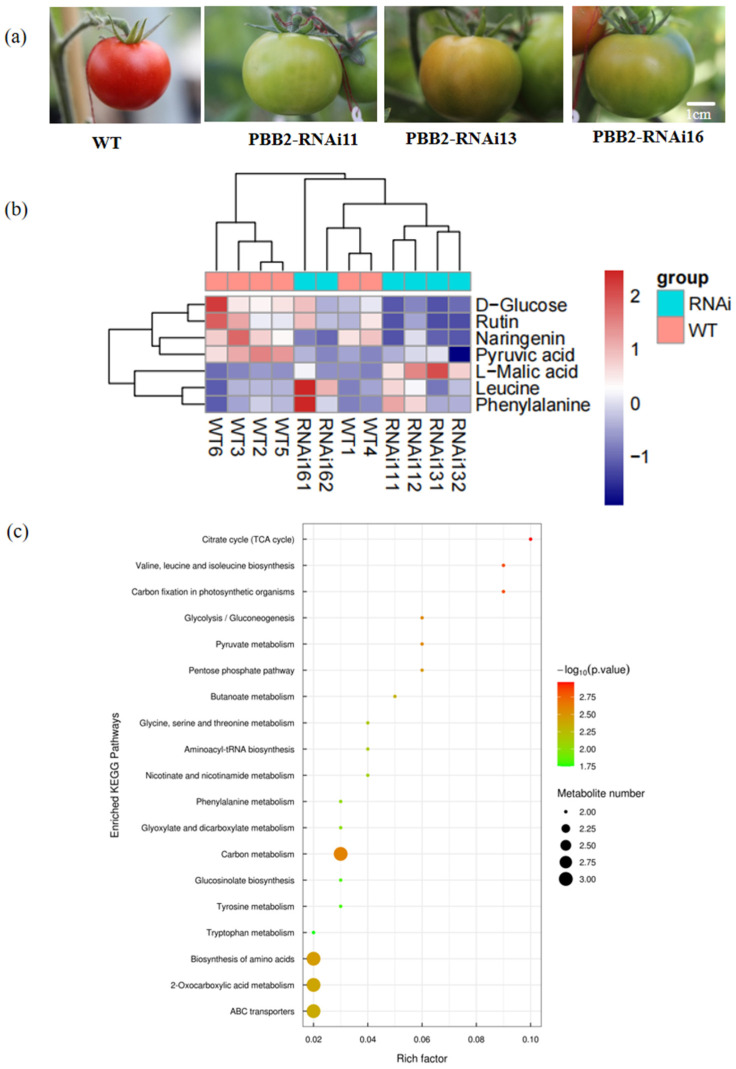
KEGG pathway annotation and enrichment analysis. (**a**) Tomato fruit phenotypic differences between Wild-Type (WT) and *PBB2*-RNAi at 43 days post-anthesis (Scale Bar = 1 cm) (**b**) Heatmap of differential metabolites clustered according to their associated KEGG pathways. Each row represents a metabolite, and each column represents a sample group. Color coding indicates upregulation (red) or downregulation (blue). (**c**) Bubble plot of enriched KEGG pathways (top 20 by significance). The *x*-axis and bubble size represent the pathway impact value from topological analysis. The *y*-axis and bubble color represent the enrichment significance (−log_10_(*p*-value)), with darker colors indicating greater significance.

The three most significantly enriched KEGG pathways were the citrate cycle (TCA cycle), carbon fixation in photosynthetic organisms, and valine, leucine, and isoleucine biosynthesis. The pathway enrichment results, presented as a bubble plot, indicate that these core metabolic processes were significantly perturbed upon *SlPBB2* silencing. KEGG pathway maps and associated heatmaps further detail the changes in metabolite levels within these pathways (Figure 7).

## 4. Discussion

In plants, the ubiquitin–26S proteasome system (UPS) serves as a crucial post-translational regulatory pathway for protein degradation. Its substrate range is exceptionally broad, encompassing nearly all aspects of the plant life cycle, including cell signaling, growth and development, circadian rhythms, and responses to biotic and abiotic stresses [14,15]. However, our understanding of UPS function, particularly its role in pigment metabolism during fruit ripening, remains limited [16,17,18].

A hallmark of fruit ripening is the transformation of chloroplasts into chromoplasts, a process involving chlorophyll degradation, thylakoid disassembly, and carotenoid accumulation [19]. The UPS regulates chloroplast proteome assembly and composition through multiple mechanisms [20]. First, within the nucleus, transcription factors such as Golden2-like (Glk) promote the expression of nuclear-encoded photosynthetic preproteins, thereby facilitating chloroplast biogenesis [21]. Second, in the cytosol, unimported preproteins are targeted for degradation by the UPS—a process mediated by the chaperone Hsc70-4 and the E3 ligase CHIP—to prevent cytotoxic aggregation [22,23]. Third, under low-gibberellin conditions, unassembled Toc159 can be degraded by the UPS following DELLA factor binding, thereby inhibiting premature chloroplast development prior to germination [24]. Finally, chloroplast-resident proteins themselves may be selectively degraded via the CHLORAD (chloroplast-associated protein degradation) system during specific developmental phases or under stress conditions [25,26,27].

Recent studies have identified several UPS components that directly regulate pigment metabolism. The plastid outer membrane-localized RING-type E3 ligase SP1 (Suppressor of ppi1 locus 1) mediates the selective degradation of plastid proteins, thereby influencing plastid transition during ripening [26]. Similarly, the E3 ligase SPL1 promotes chloroplast-to-chromoplast conversion. In tomato, the CUL4–DDB1–DET1 E3 ligase complex modulates plastid levels and pigment accumulation by targeting transcription factors such as SlGLK2 and SlBBX20 for degradation [28]. This regulatory network is further fine-tuned through the interaction of the CUL4–DDB1–DET1 complex with the methylation-recognition protein SlMBD5 [29]. Beyond tomatoes, UPS-mediated chlorophyll degradation has been documented in other fruits: In apples, the ethylene-responsive module MdPUB24–MdBEL7 regulates this process during storage [30]. In bananas, UPS-dependent chlorophyll breakdown involves multiple regulatory layers: the RING-type E3 ligase MaLUL2 contributes to chlorophyll catabolism, while high-temperature-induced “green ripening” is driven by the MaASR3–MaHDT1–MaNIP1 complex, which inhibits chlorophyll degradation through histone deacetylation [31]. Concurrently, elevated temperatures also promote MaNIP1-mediated ubiquitination and proteasomal degradation of the chlorophyll-catabolizing enzyme MaNYC1, further modulating the degreening process [32]. These findings collectively illustrate the complexity and species specificity of UPS-related regulatory networks in controlling fruit chlorophyll metabolism under varying physiological and environmental conditions.

Carotenoid accumulation is equally critical for fruit ripening. In tomato, the stability of PSY1—a key rate-limiting enzyme in carotenoid biosynthesis—is post-translationally controlled through ubiquitination by the E3 ligase PPSR1 and subsequent 26S proteasomal degradation, thereby modulating carotenoid levels [33]. In watermelons, lycopene accumulation is negatively regulated by lycopene β-cyclase (ClLCYB), which converts lycopene to β-carotene. Naturally occurring missense mutations in ClLCYB reduce protein abundance by altering its ubiquitination-mediated turnover, leading to the distinct red, yellow, and white flesh phenotypes observed in different watermelon varieties [34].

Collectively, these findings underscore the UPS as a central, multifaceted regulator of pigment metabolism during fruit ripening. It operates at multiple levels—from transcriptional regulation and protein import to the targeted turnover of metabolic enzymes—to coordinate the complex physiological transition from chloroplasts to chromoplasts. Future research should focus on elucidating the specific E3 ligase–substrate networks and signaling cascades that integrate UPS activity with hormonal and environmental cues to precisely control ripening-associated pigment changes across diverse fruit species.

## 5. Conclusions

This study employed high-resolution untargeted metabolomics to elucidate the impact of silencing the proteasome subunit gene *PBB2* on tomato fruit metabolism and quality. Our findings demonstrate that PBB2 deficiency induces significant perturbations in the tomato fruit metabolome. Key alterations were observed in the levels of primary metabolites crucial for taste and flavor (e.g., organic acids and sugars) and secondary metabolites associated with nutritional and antioxidant properties.

Bioinformatic analysis revealed that the differentially abundant metabolites were predominantly enriched in central metabolic pathways, including the citrate cycle (TCA cycle), carbon fixation in photosynthetic organisms, and amino acid biosynthesis. This indicates that PBB2 plays a pivotal role in regulating core processes such as energy metabolism, photosynthetic carbon assimilation, and nitrogen metabolism in tomato fruit. The disruption of these fundamental pathways likely underpins the observed physiological changes, including altered chlorophyll accumulation and fruit ripening dynamics.

In summary, this work establishes a critical link between the ubiquitin–proteasome system and the metabolic determinants of tomato fruit quality. By identifying specific metabolic pathways modulated by PBB2, our findings provide a novel mechanistic framework for understanding how proteasomal regulation influences fruit ripening and nutritional composition. These results offer valuable insights and a metabolic blueprint for future strategies aimed at improving tomato quality through molecular breeding or targeted cultivation practices.

## Data Availability

The original contributions presented in the study are included in the article/Supplementary Material. Further inquiries can be directed to the corresponding authors.

## Supplementary Materials

The following supporting information can be downloaded at: https://www.mdpi.com/article/10.3390/metabo16010068/s1. Table S1: Metabolite Qualitative and Quantitative Result; Table S2: Significant differential metabolites in the positive ion mode (**a**) and the negative ion mode (**b**). Figure S1: Comparison of the total ion current chromatograms (TIC) of QC samples.

## Abbreviations

The following abbreviations are used in this manuscript:

KEGG	Kyoto Encyclopedia of Genes and Genomes
UPS	Ubiquitin–proteasome system
DPA	Days post-anthesis
TIC	Total ion chromatogram
QC	Quality control
PCA	Principal Component Analysis
PLS-DA	The partial least squares–discriminant analysis
RSD	Relative standard deviation
OPLS- DA	Orthogonal projections to latent structures discriminant analysis
