# Supplementary material for: Untargeted Metabolomics Unravel the Effect of SlPBB2 on Tomato Fruit Quality and Associated Plant Metabolism
Source: Metabolites. 2026 Jan 12;16(1):68. doi: 10.3390/metabo16010068 (PMC12844362; doi:10.3390/metabo16010068)
Supplement: Supplementary file 1 [file metabolites-16-00068-s001.zip › Figure S1 Comparison of the total ion current chromatograms (TIC) of QC samples..pdf]

A

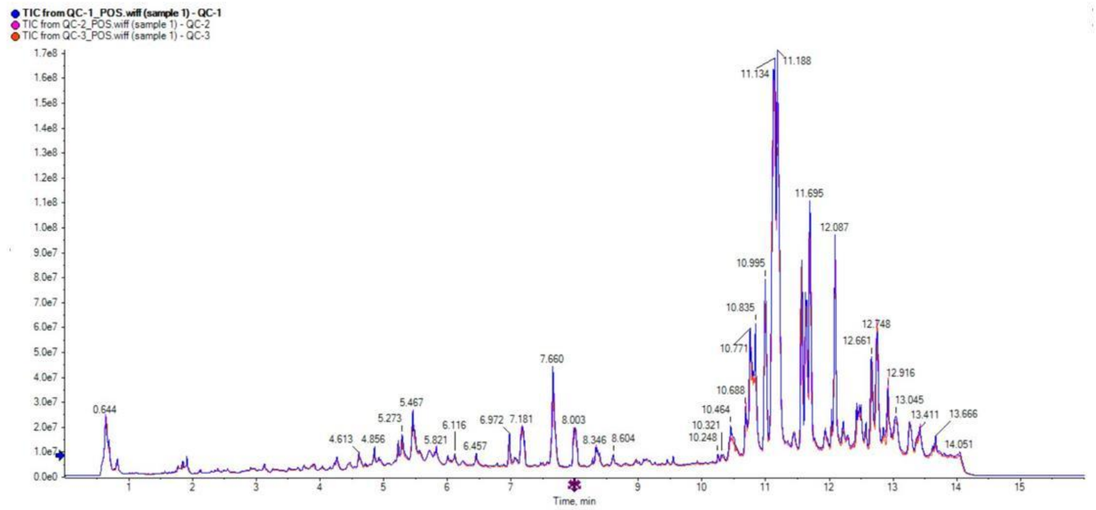

B

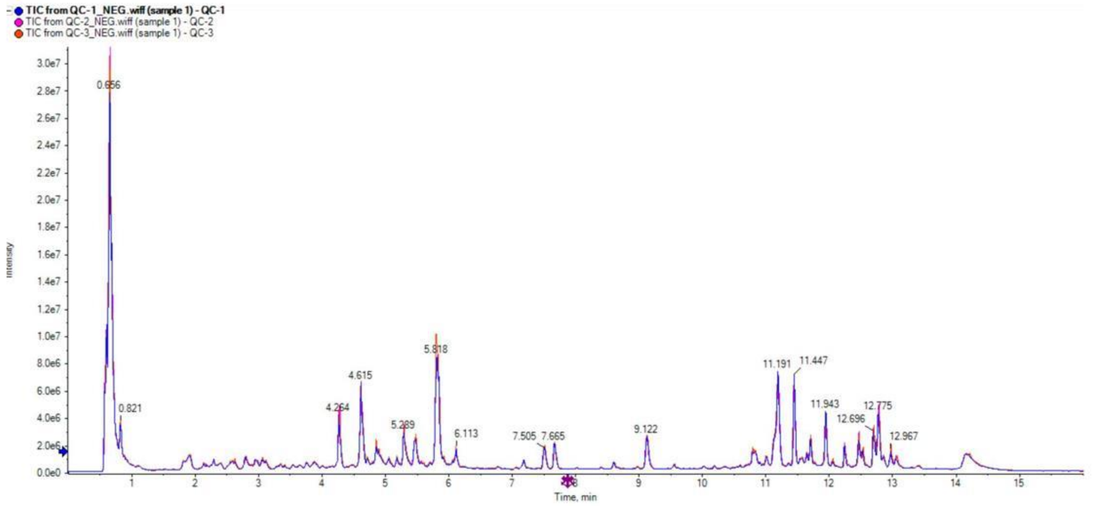

Figure S1 Comparison of the total ion current chromatograms (TIC) of QC samples. (A) Positive ion mode; (B) Negative ion mode. The horizontal axis in the figure represents the retention time of each chromatographic peak, while the vertical axis indicates the intensity value of the peak..
